# A Comparison of Clinical Outcomes between Early Cervical Spine Stabilizer Training and Usual Care in Individuals following Anterior Cervical Discectomy and Fusion

**DOI:** 10.1155/2020/5946152

**Published:** 2020-04-24

**Authors:** Carol McFarland, Sharon Wang-Price, Charles R. Gordon, Guy Otis Danielson, J. Stuart Crutchfield, Ann Medley, Toni Roddey

**Affiliations:** ^1^Texas Woman's University, School of Physical Therapy, 5500 Southwestern Medical Avenue, Dallas, TX, USA 75235; ^2^Precision Spine Care, Texas Spine and Joint Hospital, 1814 Roseland Blvd., Tyler, TX, USA 75701; ^3^Texas Spine and Joint Hospital, 1814 Roseland Blvd., Tyler, TX, USA 75701

## Abstract

**Objectives:**

Early physical therapy (PT) with specific stabilization training has been shown to benefit individuals after lumbar spinal surgery but has not been studied in patients after cervical spine surgery. The primary purpose of this study was to compare clinical outcomes between early cervical spine stabilizer (ECS) training and usual care (UC) in patients after anterior cervical discectomy and fusion (ACDF) surgery. The secondary purpose was to determine test-retest reliability of strength and endurance tests of cervical spinal stabilizers in this patient population.

**Methods:**

Forty participants who were scheduled for ACDF surgery were randomized into either the ECS group or the UC group. After surgery, participants received their assigned group intervention during their hospital stay and continued their assigned intervention for 12 weeks. All participants had phone follow-ups twice during the first 6 weeks to address questions or problems. Clinical outcome measures including pain level using the Numeric Pain Rating Scale (NPRS), disability level using the Neck Disability Index (NDI), Craniocervical Flexor Strength (CCF-S), and Craniocervical Flexor Endurance (CCF-E) were collected three times: before surgery and 6 and 12 weeks after surgery. Test-retest reliability was assessed in the first 10 participants.

**Results:**

There was no significant interaction between the groups over time for any of the outcome measures. However, all participants made significant improvements in all four outcome measures at 6 and 12 weeks post surgery. The results showed good-to-excellent test-retest reliability for the CCF-S and CCF-E tests.

**Conclusions:**

Both ECS training and UC resulted in the same amount of improvement at 6 and 12 weeks; therefore, both therapy approaches appear to have similar and positive effects on patients in their first 3 months of recovery after ACDF. Both the CCF-S and CCF-E tests can be used reliably to assess the strength and endurance of the cervical spinal stabilizers for patients after ACDF surgery. The study was registered with the ClinicalTrials.gov (NIH, U.S. National Library of Medicine, identifier # NCT01519115) Protocol Registration system.

## 1. Introduction

Physical therapy (PT) is not always a part of care following spine surgeries after patients are discharged from the hospital, although it is an integral part of care after most other orthopedic surgeries, such as total knee or total hip arthroplasty [1]. However, following spinal surgeries, patients often have difficulty returning to normal movement, can be limited by pain or fear, and are usually deconditioned due to extended periods of pain-limited activity [1–3]. It also has been shown that many individuals with spine pathologies have motor control deficits, which further decrease their capability for spinal protection during position changes or balance [4–9]. Many studies have shown that for both cervical and lumbar spine pathologies, training spinal segmental stabilizers can help with pain management, optimal spinal mobility, and functional strength [10–12]. Specific training of cervical spinal segmental stabilizers, including deep cervical flexors (DCF) and cervical multifidi (CM), has been shown to improve clinical outcomes in individuals with cervical spinal pathologies [10, 11, 13]. These smaller stabilizing muscles play a significant role in cervical spine stability, because of their ability to directly support the vertebral segments and their ability to reduce excessive pressure or stress forces on the intervertebral disc [8, 10, 11, 13].

Recent studies have shown that early PT intervention with an emphasis on motor control and spinal stabilization is beneficial for individuals after lumbar spinal surgery [12, 14–17]. Rehabilitation not only helps patients improve their motor recovery following surgery but also teaches patients nonpharmacological pain management [12, 15, 16]. It appears that the same motor control exercises and movement training, which have been shown to benefit patients with nonsurgical cervical spine pain, would also benefit patients after cervical spine surgery [11, 18, 19]. The training of these cervical spine segmental stabilizers has not been studied in individuals undergoing cervical spine surgery, even though these individuals often have problems with residual pain, weakness, and reduced postural control, similar to those found in patients with cervical spine pathology [18–21]. It is speculated that with fewer motion segments after anterior cervical discectomy and fusion (ACDF), retraining the stabilizers for optimal biomechanics is essential for full recovery of cervical spine muscle control needed for balance, functional strength, and pain management.

ACDF is one of the most common cervical spine surgeries [20–22]. In this procedure, the pathologic disc is removed almost entirely and is replaced by a spacer such as a bone graft or a cage to preserve the intervertebral space along with the foraminal opening at the level of the pathology [2, 22]. Metal plate and screw fixation is often used on the anterior vertebral bodies to secure the repair with the spacer. Although ACDF is reported to be very successful for making a structural change and generally faster recovery than lumbar surgery, patients' function is often not restored to an optimal level afterward [21, 23]. In addition, the function of segmental spinal stabilizers, such as multifidus muscle and DCF, has been shown not to return automatically without specific training after injury or structural change to the spine [8, 10, 24–26]. Therefore, motor control exercises for spinal stabilizers have been advocated in an early stage of rehabilitation [2, 9, 11, 25].

There is no consensus regarding optimal rehabilitation strategies and timing of the rehabilitation for patients who undergo ACDF. However, PT is recommended after ACDF especially for patients who still have symptoms and functional deficits. Although motor control exercises have been shown to be helpful for managing pain and dysfunction due to cervical pathologies [10, 27–30], the effectiveness of cervical spine stabilizer training has not been examined in patients after cervical surgery. Therefore, the primary purpose of the study was to examine clinical outcomes of an early cervical spine stabilizer (ECS) training starting in the hospital after ACDF surgery. Specifically, the effectiveness of ECS training vs. usual care (UC) training on pain intensity, disability level, DCF strength, and DCF endurance in patients at 6 and 12 weeks post ACDF was compared. The second purpose of the study was to establish the test-retest reliability of the Craniocervical Flexion Strength (CCF-S) test for DCF strength and CCF endurance (CCF-E) test for DCF endurance in patients post ACDF.

## 2. Materials and Methods

### 2.1. Design

This study was a prospective double-blinded randomized clinical trial. The eligible participants were randomly assigned either into the ECS training group or into the UC training group. The outcome measures were collected three times, before surgery, and at 6 and 12 weeks after ACDF. This study was approved by the principal investigator's (PI's) affiliated hospital, Texas Spine and Joint Hospital, as well as by the Institutional Review Board (IRB) at the investigators' affiliated academic institution, Texas Woman's University. In addition, this study was registered with the ClinicalTrials.gov (NIH, U.S. National Library of Medicine, identifier # NCT01519115) Protocol Registration system.

### 2.2. Participants

A power analysis was performed to estimate the adequate sample size using G∗Power 3.1 [31]. Based on the data reported in a similar study for early lumbar fusion rehabilitation [17], a small-to-medium effect size of 0.20-0.29 was estimated from their physical performance variables. Therefore, an effect size of 0.20 was used in this study to estimate the sample size. Consequently, approximately 40 participants, 20 each group, were required to achieve a power of 0.80 and an alpha level of 0.05 for a 2 × 3 repeated measure ANOVA. Participants recruited for this study consisted of those who were scheduled for ACDF surgery with one of three neurosurgeons within the same practice group. Participants were men and women between 30 and 75 years old and with preoperative diagnosis fitting in cervical pathology category III or IV from the Task Force on Neck Pain and Its Associated Disorders [32]. Cervical spine pathologies classified as category III are defined as those with neurologic deficit, such as cervical radiculopathy, without major structural pathology. Category IV pathologies are defined as those with or without neurologic deficit with major structural pathology [32]. The classification for each participant was made by their neurosurgeon, and cervical radiculopathy was verified using MR imaging and clinical examination. When a patient's cervical pathology was diagnosed with categories III and IV, an ACDF surgery was recommended by the neurosurgeon.

The exclusion criteria consisted of musculoskeletal or systemic disorders which would limit tolerance of testing or activity required for the study, pain greater than 8/10 on the Numeric Pain Rating Scale (NPRS) which may limit testing required for the study, and prior cervical spine surgeries.

Once the patients were scheduled for ACDF surgery, they were informed of this study and its risks and benefits. If they were interested in participating in this study, a study coordinator, a registered nurse, was responsible for obtaining consent from and making group assignment for each participant. Therefore, the PI who was responsible for collecting outcome measures remained blinded to group assignment throughout the study. In addition, randomization for group assignment was determined once the patients agreed to participate in the study because two informed consent forms, one for each group, were used indicating both groups would be receiving postsurgical instruction from physical therapists. Participants were blinded to the other treatment, since the separate consent forms indicated information on the specific type of instruction received. Randomization was performed by the study coordinator before surgery during preoperative testing.

After the participants signed the informed consent form, the PI completed the initial PT assessment for eligibility and enrolled the patients if cleared for surgery during the hospitalist's preoperative medical exam. The initial assessment included neurological testing as part of the preoperative evaluation to confirm the presence or absence of neurological deficits, for the participant's descriptive data. The neurological exam included sensory and motor testing of dermatomes and myotomes from C4-T1, reflex testing, Babinski's and clonus tests for compressive cord myelopathy, and upper limb tension testing for adverse mechanical neural tension.

### 2.3. Outcome Measures

The PI, a licensed physical therapist with 30 years of experience, was responsible for collecting all the outcome measures at three different times: before surgery and at 6 and 12 weeks after surgery. Outcome measures included two patient perception ratings: of pain intensity and of functional limitations. Two physical outcome measures were performed: DCF strength and DCF endurance. Pain intensity was measured using the 11-point NPRS, with 0 indicating no pain and 10 indicating unbearable pain. The functional level was determined using the 10-item Neck Disability Index (NDI) with a possible score of 0-100%, with a higher score indicating a higher disability level due to neck pain.

**Figure 1:**
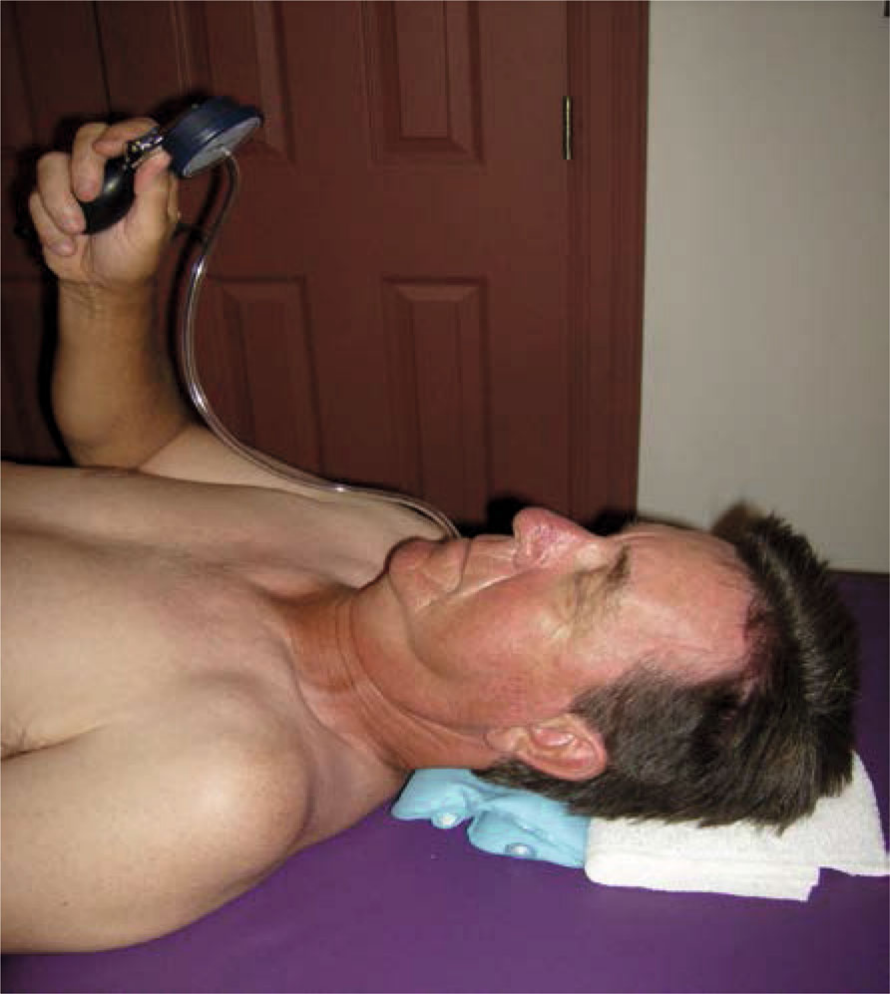
Testing position for CCF-S and CCF-E measures of DCF strength and endurance.

DCF strength was determined using the CCF-S, and DCF endurance was assessed using the CCF-E test. The CCF-S and CCF-E tests were administered using a Chattanooga Stabilizer™ Pressure Biofeedback device (DJO Ltd., UK) following the procedures described by Jull et al. [28–30] During the CCF-S test (Figure 1), the pressure biofeedback device was placed under the participant's neck and inflated to 20 mmHg while he/she was in a hook-lying supine position. The participant was asked to perform a gentle nod to increase the pressure to 22 mmHg. If the participant was able to hold the pressure with minimal muscle activation of the sternocleidomastoid and scalene muscles, the participant was asked to increase the pressure to 2 mmHg increments up to 30 mmHg. The highest pressure that the participant could hold minus 20 mmHg was the participant's CCF-S score. Therefore, the possible CCF-S score would range from 0 to 10. For the CCF-E test, the participant was asked to hold the highest pressure that the participant reached during the CCF-S test for 10 seconds 10 times with a 20-second rest period between each two repetitions. The CCF-E score is the number of times the participant could hold the pressure level for 10 seconds with correct CCF action. Therefore, the maximum score for the CCF-E test would be 100 [28]. The reliability of these two tests has been established in patients with and without neck pain but has not been established in patients after cervical spine surgery [10, 28–30]. To establish the between-day test-retest reliability of the CCF-S and CCF-E tests for this patient population, the first 10 participants, who were willing to return for the repeat testing at their 6-week follow-up, were measured on two different visits 1-2 days apart.

### 2.4. Interventions

Two hospital physical therapists who have practiced in PT for 5 and 32 years were responsible for administrating interventions for all the study participants during their hospital stay following the surgery. Prior to data collection, the PI and hospital physical therapists met and standardized the treatment protocols for both the ECS and UC groups. Patient education was emphasized during face-to-face sessions with the treating physical therapist, including written instructions and active practice of the assigned exercise and proper posture. The study coordinator informed the hospital physical therapists about the participant's group assignment, so that the PI and participants remained blinded to the group. Each participant received 1-2 PT sessions for specific group instruction during their hospital stay of 1-2 days. Once the participant was discharged from the hospital, the treating physical therapist notified the study coordinator.

The UC training of this study was the care typically provided to patients undergoing ACDF at the study hospital prior to hospital DC. The UC training consisted of patient and patient family instructions in proper head, neck, and overall spinal posture in various positions, use of cervical collar if applicable, proper body mechanics, and safety with transfers and gait. Participants in the UC training group were instructed to maintain a “head neutral” position, with an emphasis on avoiding looking up, as cervical extension avoidance is recommended for early stages after ACDF surgery. In addition, participants were instructed in nonpharmacological pain management, including ice application, deep breathing and relaxation, and the importance of walking to help the fusion and restore patient stamina. General spine surgery precautions and instructions on a DVD were given to participants so that they could review the instructions after being discharged from the hospital. This DVD was part of the study surgeons' usual care for all patients undergoing spine surgery. The DVD covered the importance of attention to spine care described above as well as gradual reconditioning, starting with walking. The information provided in the DVD is specific to the care of the spine for the first six weeks after spine surgery and is distinctly different from specific cervical spine stabilizer training. Participants were instructed to continue following the instructions up until the surgeon postsurgical follow-up at 6 weeks.

**Figure 2:**
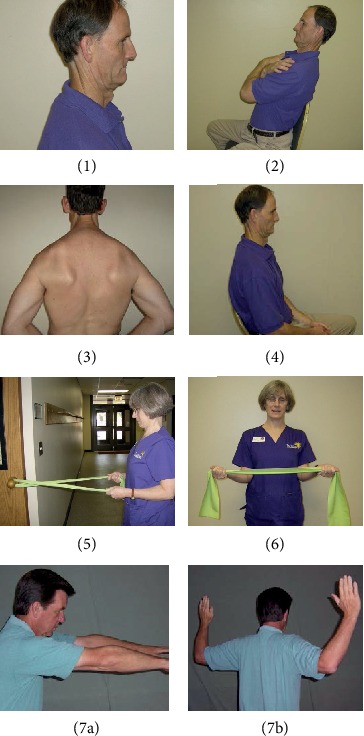
Rehabilitation research and practice submission. (1) chin tuck, (2) seated chin tuck with thoracic extension and scapular retraction, (3) standing chin tuck with scapular retraction and depression, (4) seated chin tuck with abdominal drawing in, (5) standing bilateral rows with chin tuck, (6) bilateral shoulder external rotation with chin tuck, and (7) seated reach (a) and pull (b) with chin tuck.

In addition to receiving the care described above for the UC training group, the participants in the ECS training group received specific instructions with an emphasis on safe postural muscle recruitment of the DCF as well as the synergist, CM muscles. The treating physical therapist provided detailed written descriptions with pictures for each exercise designed for achieving correct positioning and movements. The exercise program consisted of postural training in a pain-free spine-neutral position with slight chin tuck and seven exercises specifically for cervical spine stabilization as a key component of postural training (Figure 2), including (1) chin tuck and upward movement of the crown of the head (2), seated chin tuck combined with thoracic extension and scapular retraction (3), standing chin tuck combined with scapular retraction and depression (4), seated chin tuck with abdominal drawing-in (5), standing bilateral rows using a light resistance band with chin tuck, (6) bilateral shoulder external rotation using a light resistance band with chin tuck, and (7) seated reach-and-pull with chin tuck. Participants in this group were also asked to perform three additional exercises designed to improve overall functional strength, balance, and postural control (1): repeated sit-to-stands and stand-to-sits with slow descent (2), heel raises, and (3) wall slides for leg strength and overall postural cuing of the spine against the wall. The walking program was included in the exercise packet, encouraging patients to walk daily with attention to a proper posture (i.e., chin tuck and shoulder retraction). In addition, the participants in this group were instructed to increase each exercise by one repetition every other day until they could perform 30 repetitions without compensation and without aggravating symptoms. Both groups were instructed to continue their assigned program for 6 weeks and were encouraged to continue their assigned program at their 6-week follow-up.

In addition, both groups were instructed in a walking program, as surgeons often recommend for their patients during the first 6 weeks after spine surgery [1, 3]. However, the ECS group was provided in a written format and instructed to record their walking distances, whereas the UC training group was given general instructions to walk in the patient education materials given in the hospital.

### 2.5. Follow-Up

The study coordinator made all follow-up calls, using scripted questions to verify the participants' understanding of the program as well as to record their compliance. Two scheduled follow-up calls were made at two and four weeks after hospital discharge. In addition, participants were asked to call the study coordinator in the event they had questions or any problems with their assigned program. If the participants had questions regarding any part of the study or their exercises, the study coordinator relayed the information to the PI for answers without revealing the participants' name or group assignment, so that the PI could remain blinded to the participant. If needed, the study coordinator relayed the PI's answers and/or modifications of the programs to the participant. Medical or surgical questions were referred to the surgeon's office. Both groups were encouraged to contact the study coordinator with questions that occurred at any time during the study.

### 2.6. Statistical Analyses

The SPSS version 25 (IBM Corp., Armonk, NY) was used to analyze the collected data. Descriptive statistics were calculated to describe the participants' demographic and clinical examination data, including the neurologic testing results. Independent *t*-tests and chi square analysis were used to assess differences in the demographic data and baseline outcome data between groups. Four separate 2 × 3 repeated measure ANOVAs were performed to assess differences in NPRS, NDI, CCF-S, and CCF-E scores between groups at the 3 time points (before surgery and 6 and 12 weeks after surgery), respectively. Post hoc pairwise comparisons were performed when a significant interaction or the main effect was found in the ANOVAs. Lastly, the concordance correlation coefficient (*c*) was calculated to determine the between-day test-retest reliability of the CCF-S and CCF-E scores due to the homogeneity of the participants following ACDF [33]. The *α* level was set at 0.05 for all statistical analyses.

## 3. Results

**Figure 3:**
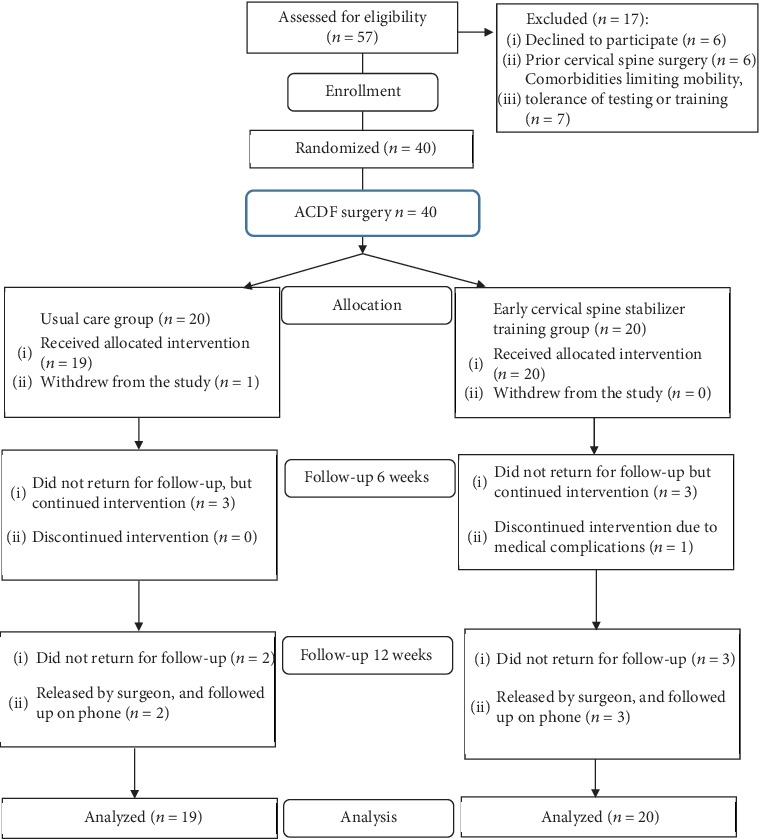
Consort diagram.

Figure 3 is a consort diagram showing participant enrollment, randomization, and dropouts. One subject in the UC group decided not to participate and withdrew from the study after enrolling. One subject in the ECS group had complications from a separate injury after the surgery and withdrew after 6-week follow-up. Thirty-nine participants, 19 in the placebo group and 20 in the ECS group, were included for the missing completely at random (MCAR) analysis. MCAR analysis was performed using SPSS on all of the collected data, including the participants' characteristics and the four outcome measures at all three time points. Because the missing data was not at random (Little's MCAR *χ*^2^ = 73.5, *P* = 0.591), imputation was performed to replace the missing data, prior to statistical analysis.

The characteristics of participants are summarized by group in Table 1. All participants were classified as being in Grade III or Grade IV of the Task Force Classifications system for neck disorders. In addition, independent *t*-tests and chi-squared analysis showed no differences in all participant characteristics at baseline (i.e., before surgery) between the two groups.

The descriptive data for the outcome measures are summarized in Table 2. The ANOVA results revealed no significant interaction between groups over time for all outcome measures: *F*_(2, 74)_ = 0.970, *η*_*p*_^2^ = 0.026, *P* = 0.355 for the NPRS; *F*_(2, 74)_ = 0.539, *η*_*p*_^2^ = 0.014, *P* = 0.585 for the NDI; *F*_(2, 74)_ = 0.018, *η*_*p*_^2^ < 0.001, *P* = 0.967 for the CCF-S; and *F*_(2, 74)_ = 0.321, *η*_*p*_^2^ = 0.009, *P* = 0.727 for the CCF-E. However, the main effect of time was significant for all outcome measures: *F*_(2, 74)_ = 56.844, *η*_*p*_^2^ = 0.606, *P* < 0.001 for the NPRS; *F*_(2, 74)_ = 54.664, *η*_*p*_^2^ = 0.596, *P* < 0.001 for the NDI; *F*_(2, 74)_ = 43.983, *η*_*p*_^2^ = 0.543, *P* < 0.001 for the CCF-S; and *F*_(2, 74)_ = 107.409, *η*_*p*_^2^ = 0.744, *P* < 0.001 for the CCF-E. Post hoc pairwise comparisons showed significant improvements (*P* < 0.001-*P* = 0.008) in all outcome measures from before surgery to 6 weeks after surgery and from 6 weeks after surgery to 12 weeks after surgery, except for the NPRS scores from 6 weeks after surgery to 12 weeks after surgery (*P* = 0.364). In summary, NPRS, NDI, CCF-S, and CCF-E results tested before surgery showed all participants exhibited a moderate pain level and disability level with 50% deficit, CCF-S with 40% deficit, and CCF-E with 75% deficit. At the end of the 12 weeks, the participants still had approximately 20% deficits of pain, DCF strength, and endurance. Lastly, eight of the ten planned participants were able to complete the test-retest reliability part of the study for the CCF-S and CCF-E tests on both days. The concordance reliability analysis showed good between-day test-retest reliability (*ρ*_*c*_) for the CCF-S (*ρ*_*c*_ = 0.82) and CCF-E (*ρ*_*c*_ = 0.70) tests.

## 4. Discussion

The results showed no significant differences in the pain level, disability level, DCF strength, and DCF endurance between groups at 6 weeks and 12 weeks after surgery. The significant improvements by time in both approaches could be due to both groups receiving the same amount of face-to-face instructions during their hospital stay after surgery and the same amount of follow-up phone calls, which provided equal opportunities for both groups to ask questions regarding their rehabilitation programs. The study also assumed that 1-2 sessions of face-to-face instructions and phone follow-ups would be adequate to instruct DCF training, a type of motor control exercise, for the ECS group. Given that the literature supports the need for feedback and repetition for learning with motor control exercise, lack of face-to-face instructions may discount the treatment effects for this group [5, 9–11].

In addition, it is uncertain how much proper posture, which was instructed to the UC training group, could have affected DCF muscle activation. Both groups showed improvement in DCF recruitment with CCF-S and CCF-E tests at 6 and 12 weeks. Proper posture instruction often leads to a chin-in motion, which could have activated the DCF in the same manner as the exercises performed by the ECS training group. Lastly, recorded instructions have shown to improve exercise adherence, which in turn impacts rehabilitation outcomes [34, 35]. All participants received a DVD of the proper posture and beneficial activity after spine surgery, which could have further equated the two intervention approaches [36].

A recent study by Coronado et al. [41] examined the feasibility and outcomes of an early self-directed home exercise program after ACDF. Similar to the results of our study, they did not find significant differences in their outcome measures between the early rehabilitation program and UC. However, Coronado et al. found the early rehabilitation program to be safe for short-term pain management. Although our study examined the addition of motor control exercise to the early rehabilitation for the patients who underwent ACDF, our results also support the use of early intervention following ACDF surgery.

The results of this study were different from those of early spinal stabilization exercises on patients after lumbar spinal surgery [12, 14–17]. The difference in these studies from our study is the distinct contrast between the lumbar studies' experimental and control groups, since there was no PT in their control groups. In this study, both groups had PT intervention with either early PT ECS training or UC training. In addition, the early PT intervention in the lumbar spine studies often included multiple sessions of one-on-one instruction with feedback from treating therapists, including the use of a pressure biofeedback to ensure activation of the lumbar spine stabilizers and supervised sessions for exercise progression [17]. Further, a multimodal approach (i.e., early lumbar spinal stabilizer training combined with behavior or cognitive therapy) was included in several of these studies as behavioral or cognitive training has been shown to help improve recovery from spine surgery [12, 37, 38]. Given recent motor control studies specify 8 or more weeks necessary to integrate strength changes with spine stabilizers, so that 12 weeks may have not been a long enough follow-up time to see differences between groups [5, 10, 18]. Differences between groups regarding long-term recovery and postsurgical medication use are unknown.

All the participants made significant improvements in the pain level, disability level, DCF strength, and DCF endurance at 6 weeks after surgery and continued making improvement from 6 weeks after surgery to 12 weeks after surgery. As with any postsurgical rehab study, the improvement could be attributed to surgery alone. As noted in the NPRS, NDI, CCF-S, and CCF-E results for all participants before surgery, pain and disability testing showed 50% deficit, CCF-S with 40% deficit, and CCF-E with 75% deficit. At the end of the 12 weeks, the participants still had approximately 20% deficits of pain, DCF strength, and endurance. Without long-term follow-ups, the ultimate outcome is not certain. However, it has been shown that when patients make improvements in pain and disability (i.e., exceeding MCID) after spine surgery in the first three months, they are likely to continue to improve and have successful clinical outcome at one year [39]. Part of the therapy role is to maximize the long-term follow-up with reconditioning functional strength, mobility, and postural control of the upper body. In addition, with therapy, patients can learn the most effective nonpharmacological pain management. The lumbar spine studies had longer follow-up periods and emphasized the effectiveness of long-term training [12, 16, 17]. Interestingly, although DCF endurance appeared to be affected the most before surgery in this study, the participants improved in DCF endurance the most in the first 3 months. Therefore, follow-up longer than 3 months would be warranted for future studies to examine outcomes of cervical spine surgery and rehabilitation.

The results of the reliability analysis also establish an excellent between-day test-retest reliability for the CCF-S and CCF-E tests on patients after ACDF [33]. The results are similar to the results found by Fernandez-de-la-Penas et al. who reported ICCs of 0.84-0.9 for patients with chronic tension-type headaches [30] and by Chiu et al. [29] who reported 80% agreement between testers for patients with neck pain. The concordance correlation coefficient of the CCF-E test was slightly lower than that of the CCF-S, possibly due to the variation in the determination of performance failure at each stage of CCF-E. In summary, both the CCF-S and CCF-E tests can be useful clinical assessment tools for monitoring patients after a cervical surgery.

### 4.1. Study Limitations

There were several limitations in this study. Participants had variable preoperative diagnoses, which may have led to different responses to surgery and outcome. In addition, there was a range of neurological symptoms among the participants, which may have affected outcomes. For example, neurologic motor or sensory changes into the upper extremities increase the factors influencing the pain level or functional score. Further, the ACDF surgeries were performed by three different surgeons. Although all three surgeons practiced in the same group and had similar surgical training, there still could be variations in the surgical procedures. As discussed above, participants had only 1-2 sessions of face-to-face PT sessions, learning was dependent on the use of written and DVD instructions, and feedback was available only via phone follow-ups and retesting visits. The limited time with the physical therapists may not have been adequate for participants to learn and respond to early intervention training and motor learning. Lastly, exercise adherence was not tracked for the either group. As shown in the literature, exercise adherence is poor after discharge from PT. [40] Poor adherence could reduce the patient participation in their recovery following ACDF. Patient engagement in their recovery has been shown to improve outcomes in many previous spine surgery studies [12, 14, 16, 41]. Although we are not certain if participants had poor exercise compliance, two follow-up calls were made to all participants at two and four weeks after hospital discharge to inquire about their performance of the assigned PT program. In addition, the participants gave verbal feedback about following their group instructions. This was reported to the PI at follow-up testing at 6 and 12 weeks, which was general nonmeasurable information, especially since the PI was blinded to the group.

## 5. Conclusions

Both CCF strength and endurance tests are reliable when used for patients after ACDF surgery. Because both the UC training and ECS training resulted in the same amount of improvements at 6 and 12 weeks after ACF surgery, UC training without emphasis on DCF training may be sufficient for the patient's first three months of recovery after ACDF surgery. Future studies should include more differentiation between training groups, more testing of specific motor control exercises as part of PT, and a follow-up longer than three months to monitor the progression of the patient population.

## Figures and Tables

**Table 1:** Characteristics of the participants in the usual care (UC) group and the early cervical spine stabilizer (ECS) training group prior to surgery.

	UC (*n* = 19)	ECS training (*n* = 20)	*P* value
Age (years)	56.0 ± 9.8	54.7 ± 10.5	0.582
Gender (female/male)	9/10	14/6	0.200
Neurological exam (number)			
Unilateral radicular pain	11	10	0.621
Bilateral radicular pain	3	6	0.451
Motor deficit	11	12	0.894
Sensory deficit	9	8	0.68
+Upper limb tension test	15	14	
Cervical spine task force			
Classification category III/IV	14/5	10/10	0.129
Number of levels fused^∗∗^	8/11	10/10	0.621
(One/two)			

^∗^ *P* < 0.05 for independent *t*-test or chi-squared test. ^∗∗^Surgeries were all ACDF confirmed during the study by operative report and postsurgical radiographs.

**Table 2:** Means and standard deviations of the NPRS, NDI, CCF-S, and CCF-E scores for all participants, the usual care (UC) group, and the early cervical stabilizer (ECS) training group.

	All (*n* = 38)	UC (*n* = 19)	ECS training (*n* = 20)	*P* value for interaction
NPRS				0.355
Before surgery	5.3 ± 2.4	5.1 ± 2.7	5.6 ± 2.2	
At 6 weeks	2.1 ± 1.9	2.1 ± 1.5	2.0 ± 2.2	
At 12 weeks	1.9 ± 1.6	2.2 ± 1.6	1.7 ± 1.7	
NDI				0.583
Before surgery	46.8 ± 17.3	48.2 ± 19.0	45.4 ± 16.0	
At 6 weeks	28.3 ± 16.1	32.2 ± 14.2	24.6 ± 17.2	
At 12 weeks	20.5 ± 14.6	24.2 ± 12.0	16.9 ± 16.1	
CCF-S				0.967
Before surgery	6.2 ± 2.3	6.0 ± 2.5	6.4 ± 2.1	
At 6 weeks	8.4 ± 1.7	8.2 ± 2.0	8.6 ± 1.3	
At 12 weeks	9.1 ± 1.5	9.0 ± 1.7	9.3 ± 1.3	
CCF-E				0.727
Before surgery	30.7 ± 17.1	29.1 ± 19.9	32.3 ± 14.4	
At 6 weeks	66.0 ± 20.0	61.3 ± 20.9	70.4 ± 18.5	
At 12 weeks	85.7 ± 21.1	81.7 ± 23.3	89.4 ± 18.6	

^∗^ *P* values for interactions of 2 × 3 repeated measure ANOVAs. NPRS: Numerical Pain Rating; NDI: Neck Disability Index; CCF-S: Craniocervical Flexion Strength test; CCF-E: Craniocervical Flexion Endurance test.

## Data Availability

This study is not including data availability, due to limitations of medical records privacy requirements of hospital and study surgeons.
